# Circulating chemerin levels in preeclampsia: a systematic review and meta-analysis

**DOI:** 10.1186/s12944-023-01941-w

**Published:** 2023-10-20

**Authors:** Yangxue Yin, Shuangshuang Xie, Qin Xu, Lingyun Liao, Hongqin Chen, Rong Zhou

**Affiliations:** 1 Department of Obstetrics and Gynecology, West China Second University Hospital, Sichuan University, Key Laboratory of Birth Defects and Related Diseases of Women and Children (Sichuan University) of Ministry of Education, No. 20, section 3, Renmin South Road, Wuhou District, Chengdu, Sichuan 610041 China; 2 NHC Key Laboratory of Chronobiology, Sichuan University, Chengdu, China

**Keywords:** Preeclampsia, Chemerin, Adipokine, Systematic review, Meta-analysis

## Abstract

**Background:**

Preeclampsia (PE) is a new-onset pregnancy-specific disorder with a high prevalence that leads to over 70 000 maternal and 500 000 foetal fatalities worldwide each year. The level of chemerin, a newly identified adipokine, is increased in diabetic and obese patients. Currently, there are several studies describing the relationship between maternal circulating chemerin levels and PE. Therefore, this study aimed to assess their association in pooled samples.

**Methods:**

Four databases were systematically searched to identify potential studies that reported circulating chemerin levels in PE and normal pregnancy groups. Standardized mean differences (SMDs), 95% confidence intervals (CIs), and 95% prediction intervals (PIs) were calculated using a random-effects meta-analysis. The probability of heterogeneity was also investigated by sensitivity analysis, subgroup analysis, and meta-regression.

**Results:**

Thirteen studies in 11 articles with a total of 860 PE patients and 1309 women with normal pregnancies met the inclusion criteria. The results of the meta-analysis revealed that circulating chemerin, which levels in PE patients were considerably higher than those in controls (SMD = 1.39, 95% CI: 1.02, 1.77, 95% PI: -0.07, 2.86). Moreover, sensitivity analysis determined that the outcomes of the overall pooled results were not affected after the elimination of any study. Notably, subgroup analysis demonstrated a similar expression pattern irrespective of geographic location, severity, timing of sampling, and sample size. Last, there were no factors that significantly impacted the overall estimate, according to meta-regression.

**Conclusions:**

This meta-analysis is the first to assess circulating chemerin levels in PE patients. The findings indicate that circulating chemerin levels may be a potential marker to diagnose PE.

**Supplementary Information:**

The online version contains supplementary material available at 10.1186/s12944-023-01941-w.

## Introduction

Preeclampsia (PE) is a new onset pregnancy-specific disorder that occurs after 20 weeks of gestation with an estimated incidencerate of 3-5%, characterized by hypertension and accompanied by proteinuria and/or systemic multiorgan dysfunction [1]. Its clinical manifestations are intricate and extremely dangerous for mothers and children, leading to over 70 000 maternal and 500 000 foetal fatalities each year [2]. PE can be classified as mild PE or severe PE depending on its progression and can trigger grave complications, such as cerebrovascular disorders, liver rupture, pulmonary oedema, renal failure, intrauterine growth restriction, preterm birth, and intrauterine death if not promptly treated [3]. In addition, the long-term effects of PE are a higher risk of future cardiovascular disorders in mothers and their offspring [4–6]. The placenta is involved in the pathogenesis of PE, so delivery of the placenta may be the only effective treatment [7]. Although the pathogenesis of PE remains obscure, there is growing evidence that its clinical symptoms are exacerbated by insufficient spiral artery remodelling, resulting in placental hypoxia, immune activation, the generation of proinflammatory cytokines, reduced levels of proangiogenic factors, and elevated levels of antiangiogenic factors [8].

Despite the existence of a gold standard for the diagnosis of PE, the discovery of several biomarkers in easily accessible samples (such as blood and urine samples) can enhance the early diagnosis and prediction of PE, as well as provide insights into its pathogenesis [9]. At present, numerous evidence-based guidelines for PE recommend the utilization of angiogenic biomarkers, of which the most thoroughly studied is the soluble fms-like tyrosine kinase-1 (sFlt-1) / placental growth factor (PlGF) ratio. For patients with a clinical diagnosis of PE, the ratio can assist in more accurate diagnosis or classification, and a ratio ≤ 38 can be utilized to predict the short-term absence of illness [10, 11]. However, there is no single biomarker that can accurately predict or diagnose PE, and consequently, further exploration of other reliable biomarkers is warranted to intervene early in the development of the disease and improve pregnancy outcomes.

Adipokines are autocrine and paracrine molecules that are secreted by adipose tissue and execute various biological functions. Chemerin, a novel adipokine, initially linked to psoriasis, is now known to be involved in inflammation, adipogenesis, angiogenesis, and energy metabolism [12, 13]. Numerous prospective studies have established a substantial positive correlation between various diseases and chemerin levels independent of established risk factors, such as cardiovascular disease and colorectal cancer [14, 15]. In PE patients, chemerin appears to inhibit trophoblast migration, invasion, tube formation and disrupt trophoblast lipid metabolism [16, 17]. Hence, it was hypothesized that circulating chemerin levels in pregnant women may be associated with the development of PE and may be a convenient and cost-effective screening and diagnostic biomarker.

Under such circumstances, several studies have evaluated the expression of circulating chemerin in normal pregnancy and PE groups. According to the group’s review of the literature, no previous meta-analyses have quantitatively summarized the relationship between chemerin and PE. In this study, a systematic review of original research was conducted to incorporate recent data from studies on chemerin and elucidate the relationship between this special biomarker and PE.

## Methods

This study was registered in PROSPERO (No: CRD42023430965).

### Search strategy

Two researchers (Y Yin and S Xie) independently searched English databases, including PubMed, Embase, Web of Science, and Cochrane Library, for articles published up to Sep 4, 2023. The following retrieval terms were employed to access relevant data: ((“Pre-Eclampsia” [MeSH] OR preeclampsia OR PE OR “Hypertension, Pregnancy-Induced” [MeSH] OR “hypertensive disorders of pregnancy” OR “gestational hypertension”) AND chemerin). The specific search strategy is shown in Supplemental Table 1. Moreover, the references of the selected articles were manually screened to avoid omissions. Any discrepancies were resolved by arbitration with a third researcher (Q Xu).

### Inclusion and exclusion criteria

Observational studies assessing the relationship between chemerin levels and PE were eligible for inclusion in this study.

The inclusion criteria were as follows: (1) articles published in peer-reviewed journals that reported on circulating chemerin levels in PE and normal pregnancy groups; (2) articles published in English; and (3) studies in which PE (both mild and severe PE) was diagnosed based on generally accepted guidelines, where mild PE was defined as PE without severe features, and severe PE was defined as PE with at least one severe features (blood pressure over 160/110 mmHg, thrombocytopenia, impaired liver function, renal insufficiency, pulmonary oedema, new-onset headache not accounted for by alternative diagnoses, and visual disturbances); and studies in which PE have been described based on the timing of disease onset: early-onset PE occurring < 34 weeks’ gestation and late-onset PE occuring ≥ 34 weeks’ gestation [18, 19].

The exclusion criteria were as follows: (1) reviews, commentaries, or conference abstracts; (2) studies of PE superimposed on other medical conditions; and (3) cellular or animal studies without clinical data.

### Data extraction

Two researchers (Y Yin and L Liao) screened the articles by the inclusion and exclusion criteria. First, by reading the titles and abstracts, duplicates and totally irrelevant studies were eliminated. Subsequently, the full texts were read, and eligible studies were included. Disagreements were resolved by Q Xu. The following data were extracted: first author, publication year, country, study design, specimen type, detection method, classification of PE, measurement before or after PE diagnosis, gestational weeks at sampling, sample size, chemerin concentration, maternal age, body mass index (BMI), sensitivity, specificity, cut-off values, and area under the curve (AUC).

### Quality assessment

The quality of the selected studies was evaluated using the ROBINS-E tool [20]. Quality assessment was performed independently by Y Yin and H Chen, and discrepancies were resolved by arbitration with a third researcher (Q Xu). Seven domains were covered: (1) bias due to confounding; (2) bias in selecting participants in the study; (3) bias in exposure classification; (4) bias due to departures from intended exposures; (5) bias due to missing data; (6) bias in outcome measurement; (7) bias in the selection of reported results. In addition, the overall credibility of evidence was assessed using the GRADE approach [21].

### Statistical analysis

Statistical analyses were conducted using Stata 15.0 (StataCorp LP, College Station, TX, USA). Variables are presented as the mean ± standard deviation (SD) and summary measures are presented as standardized mean differences (SMDs) with 95% confidence intervals (CIs) and 95% prediction intervals (PIs). Data expressed as the median and range or interquartile range (IQR) were converted to the mean ± SD [22, 23]. The majority of studies reported two subgroups, namely mild and severe PE subgroups [24–26], and they were combined so that each study could contribute a single effect size estimate [27]. The formulae were as follows:

$$\begin{gathered}Mean = (N1M1 + N2M2)/(N1 + N2) \hfill \\SD = \sqrt {\frac{{(N1 - 1)SD{1^2} + ({N_2} - 1)S{D_2}^2 + \frac{{{N_1}{N_2}}}{{{N_1} + {N_2}}}({M_1}^2 + {M_2}^2 - 2{M_1}{M_2})}}{{{N_1} + {N_2} - 1}}} \hfill \\ \end{gathered}$$

(N = sample size, M = mean of the group)

Higgins’ *I*-square test based on Cochrane’s *Q* was used to assess statistical heterogeneity among the included studies, and an *I*^2^ value of > 50% was regarded to indicate significant heterogeneity. The random-effects model (DerSimonian and Laird) was applied in this study [28]. Sensitivity analysis was used to identify sources of heterogeneity. Publication bias was examined qualitatively by funnel plot and quantitatively by Egger’s test, Begg’s test, and the trim and fill method. Subgroup analysis was conducted based on geographical location, PE severity, timing of sampling, and sample size to explore possible sources of heterogeneity. Meta-regression was performed on the geographical location, timing of sampling, and sample size. A *P* value < 0.05 was considered statistically significant.

## Results

### Study selection

**Fig. 1:**
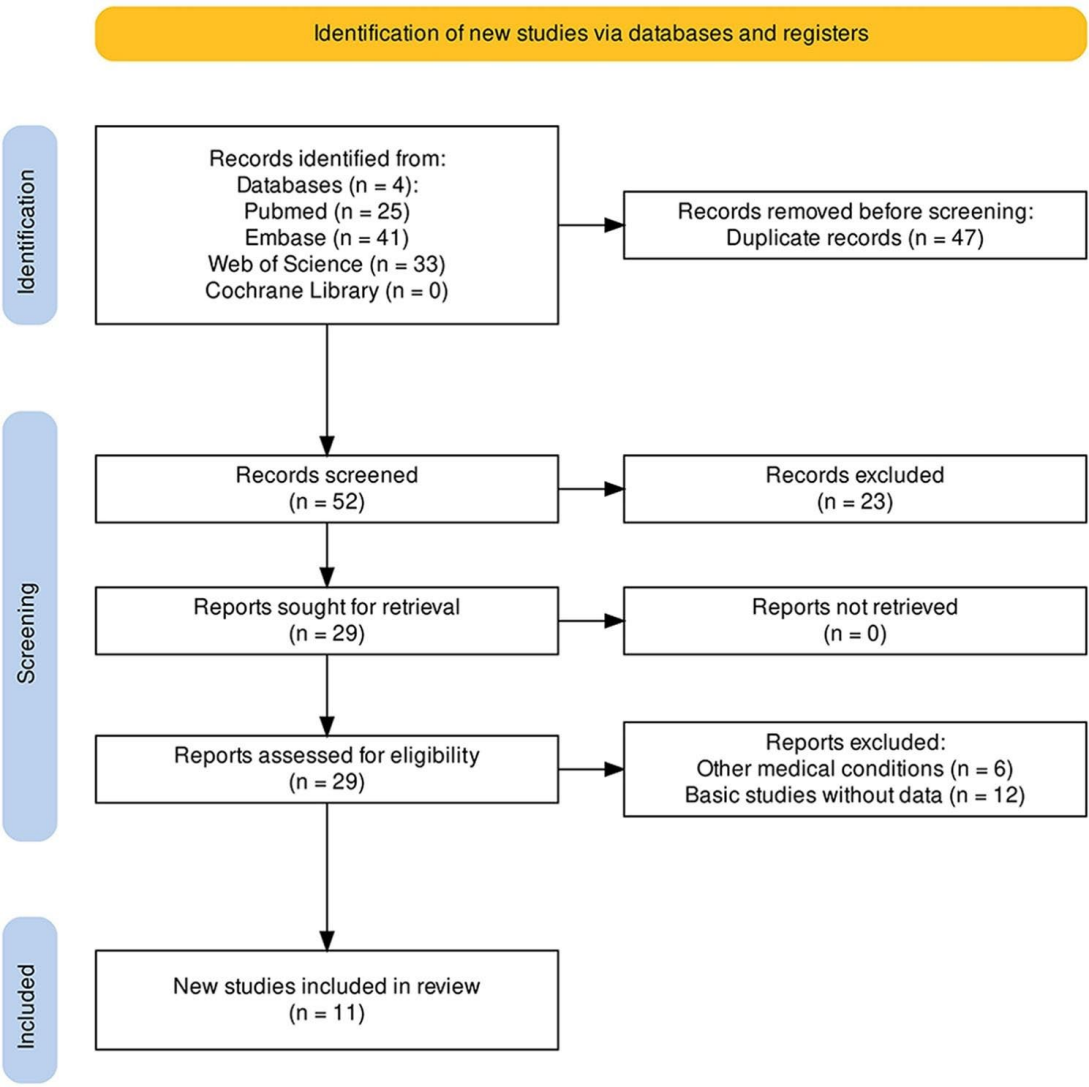
Flowchart of study selection

Ninety-nine articles were identified through the search strategy. After removing duplicates, 52 articles remained. Twenty-three articles were subsequently excluded by reading the titles and abstracts, largely owing to the studies being reviews, commentaries, or conference abstracts. After retrieval of the articles, 18 articles were subsequently excluded from the remaining 29, chiefly due to the presence of other medical conditions (n = 6) and cellular or animal experiments without available clinical sample data (n = 12). Ultimately, 11 articles were included in the study [24–26, 29–36] (Fig. 1).

### Study characteristics

Across the 13 studies in the 11 articles, a total of 2169 participants were included, among whom 1309 had normal pregnancies and 860 had PE patients (Table 1). In terms of geographic locations, 9 studies were conducted in Asia, 2 were conducted in Oceania, 1 was conducted in Europe, and 1 was conducted in Africa. In 2 of the 13 studies, blood samples were collected before the diagnosis of PE. Three studies evaluated the accuracy (sensitivity, specificity, and cut-off) of circulating chemerin levels in distinguishing pregnancies with PE from normal pregnancies, one of which yielded results before diagnosis. In addition, 2 studies only reported AUCs. Based on ROBINS-E tool, one study had a serious risk of bias, and the rest of the studies were evaluated to have a moderate risk of bias (Supplementary Table 2). The GRADE approach suggested that this meta-analysis had very low credibility of the overall evidence due to the observational nature of the study, serious risk of bias, serious inconsistency, and other plausible confounding (Supplementary Table 3).

**Table 1:** Characteristics of the included studies evaluating the relationship between circulating chemerin levels and preeclampsia

No	First author	Year	Country	Study design	Specimen type	Detection method	Before/after PE diagnosis	Classification of PE	GA at sampling (weeks)		Sample size		Chemerin (ng/ml)		Age (years)		BMI (kg/m^2^)		Sen (%)	Spe (%)	Cut-off (ng/mL)	AUC
									control	PE	control	PE	control	PE	control	PE	control	PE				
1	Stepan	2011	Germany	Cross-sectional	Serum	ELISA	After	Total	28.86 (25.57–37) ^a^	29.57 (23.86–39.86) ^a^	37	37	205.82 ± 33.48	248.55 ± 57.36	30 (18–40) ^a^	31 (19–39) ^a^	22.0 (18.4–43.0) ^a^	22.4 (16.9–31.2) ^a^	NA	NA	NA	NA
2	Al-Refai	2012	Saudi Arabia	Cross-sectional	Serum	ELISA	After	Total	33.76 ± 3.29	32.68 ± 4.88	30	29	174.40 ± 29.17	349.90 ± 147.92	31.96 ± 5.27	34.37 ± 5.79	29.36 ± 2.69	30.46 ± 1.3	NA	NA	NA	NA
								Mild				19		281.95 ± 144.54								
								Severe				10		479 ± 158.56								
3	Duan	2012	China	Cross-sectional	Serum	ELISA	After	Total	36.38 ± 3.59	35.83 ± 3.42	72	72	210.80 ± 47.34	258.85 ± 86.64	28.83 ± 4.32	29.19 ± 5.06	20.83 ± 2.75 ▲	23.24 ± 3.31 ▲	NA	NA	NA	NA
								Mild				49		228.1 ± 87.99								
								Severe				23		289.6 ± 74.43								
4	Xu	2014	China	Prospective cohort	Serum	ELISA	Before	Total	10.64 ± 0.61	10.67 ± 0.49	477	41	181.4 ± 78.6	312.1 ± 112.7	25.0 ± 4.7	27.2 ± 4.4	22.6 ± 2.5 ▲	25.4 ± 2.6 ▲	87.8	75.7	183.5	0.766
								Mild				23		270.3 ± 91.8								
								Severe				18		365.5 ± 116.5								
5	Turgut	2015	Turkey	Cross-sectional	Serum	ELISA	After	Total	35.5 (26–38) ^b^	34.5 (25–40) ^b^	32	32	163.39 ± 13.77	218.84 ± 17.40	31.03 ± 7.12	31.28 ± 8.05	24.5 (21.2–24.9) ^b^▲	24.6 (20.8–24.9) ^b^ ▲	NA	NA	NA	NA
6	Wang	2015	China	Cross-sectional	Serum	ELISA	After	Total	39.3 ± 0.9		28	60	220.00 ± 50.78		28.2 ± 4.8		26.28 ± 3.16		NA	NA	NA	NA
								Mild		38.5 ± 1.6		30		330.23 ± 56.22		27.9 ± 6.5		27.49 ± 2.68				
								Severe		37.4 ± 2.9		30		493.83 ± 105.23		28.5 ± 5.5		28.65 ± 3.98				
7	Cetin	2017	Turkey	Cross-sectional	Serum	ELISA	After	Total	NA		46	88	199.96 ± 28.05		26.59 ± 3.68		25.62 ± 2.24		95.5	95.7	252	NA
								Mild		NA		45		322.11 ± 37.60		28.11 ± 5.02		26.86 ± 1.93				
								Severe		NA		43		394.72 ± 100.01		27.65 ± 6.64		26.10 ± 2.35				
8	Murad	2020	Iraq	Cross-sectional	Serum	ELISA	After	Total	NA		34	66	202.6 ± 21.1		26.23 ± 6.5		29.35 ± 4.1		89.3	94.1	228.5	NA
								Mild		NA		33		227.49 ± 57.4		25.0 ± 5.0		26.61 ± 3.8	NA	NA	NA	NA
								Severe		NA		33		435.06 ± 55.4		24.45 ± 6.3		28.43 ± 3.8	93.9	100	380.9	NA
9	Tan	2022	China	Cross-sectional	Serum	ELISA	After	Total	34 (28.86–36.29) ^b^	32.29 (27–36.14) ^b^	29	30	156.85 ± 87.33	285.93 ± 130	33 (28–37) ^b^	32 (30–36) ^b^	25 (23–31) ^b^ ▲	25 (22–28) ^b^▲	NA	NA	NA	NA
10	Bartho (1)	2023	Australia	Cross-sectional	Plasma	ELISA	After	Early-onset	26.38 ± 0.98	29.65 ± 0.39	17	46	346.39 ± 137.42	487.71 ± 176.01	32.63 ± 0.95	30.83 ± 0.84	24.25 (28.93–20.55) ^b^	28.50 (35.20–26.00) ^b^	NA	NA	NA	0.75
11	Bartho (2)	2023	South Africa	Cross-sectional	Plasma	ELISA	After	Severe	NA	NA	15	26	111.82 ± 44.98	183.71 ± 94.12	NA	NA	NA	NA	NA	NA	NA	NA
12	Bartho (3)	2023	Australia	Prospective cohort	Plasma	ELISA	Before	Total	36.14 (35.71–36.57) ^b^	36.28 (35.57–36.57) ^b^	182	23	86.05 ± 20.18	111.08 ± 29.24	32 (29.75–35) ^b^	34 (32–37) ^b^	24.55 (22.29–28.12) ^b^	27.88 (24.14–30.66) ^b^	NA	NA	NA	0.8
13	Chen	2023	China	Cross-sectional	Serum	ELISA	After	Total	35.0 (34.0–36.0) ^b^	35.0 (34.0–36.0) ^b^	310	310	140.2 ± 53.5	171.80 ± 49.20	33.5 (31.0–36.0) ^b^	33.0 (30.0–36.0) ^b^	23.0 ± 3.3 ▲	22.5 ± 3.4 ▲	NA	NA	NA	NA

PE: preeclampsia; GA: gestational age; BMI: body mass index; Sen: sensitivity; Spe: specificity; AUC: area under curve; NA: not available

^a^ Median (range); ^b^ Median (interquartile range); ▲ Pre-pregnancy BMI

### Synthesis of results

**Fig. 2:**
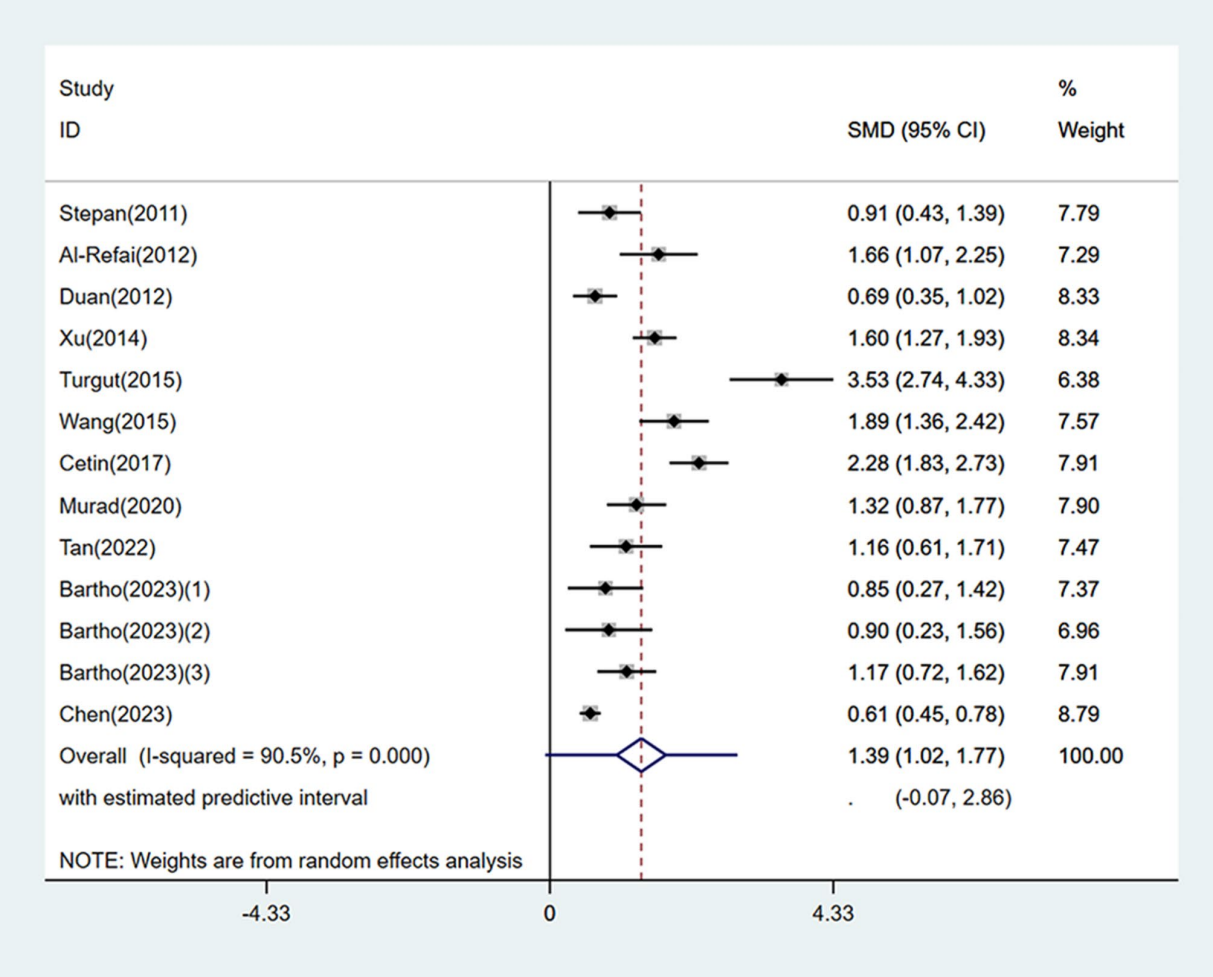
Forest plot of circulating chemerin levels in preeclampsia patients compared to controls

A meta-analysis was performed on the 13 studies using the random effects model, and the pooled results revealed that circulating chemerin levels were significantly higher in the PE group than in the control group (SMD = 1.39, 95% CI: 1.02, 1.77, 95% PI: -0.07, 2.86). Nevertheless, heterogeneity was high (*I*^*2*^ = 90.5%, *P* < 0.001) (Fig. 2).

### Sensitivity analysis and publication bias

**Fig. 3:**
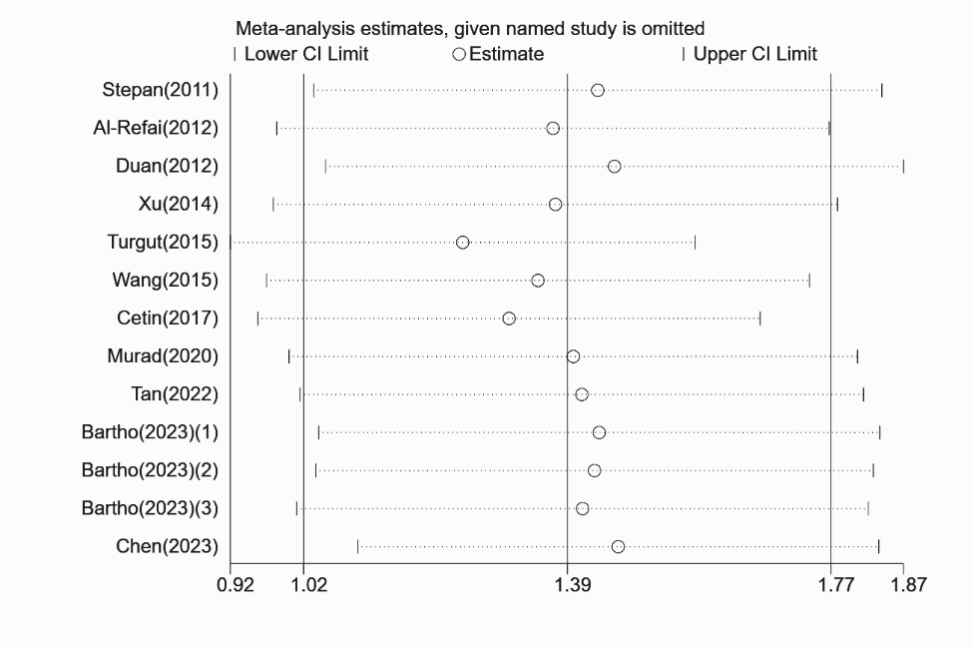
Sensitivity analysis

**Fig. 4:**
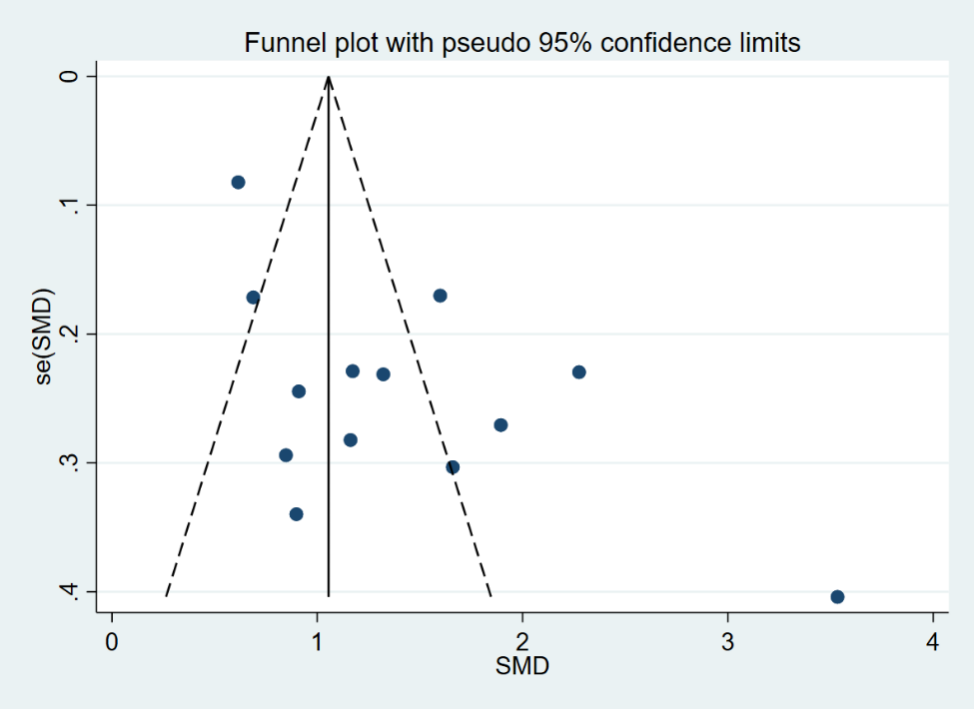
Funnel plot of the variance in circulating chemerin levels for the visual identification of publication bias

Sensitivity analysis uncovered that the outcomes of the overall pooled results were not affected by the elimination of any study, indicating that the results were robust, and consequently, no studies were excluded (Fig. 3). The presence of publication bias was determined qualitatively by visualizing the funnel plot, which was asymmetric (Fig. 4). Similarly, the Egger’s test quantitatively validated the asymmetry of the funnel plot (*P* = 0.01). However, the Begg’s test did not suggest a presence of publication bias, and there may be other reasons for the asymmetry of the funnel plot (*P* = 0.428). Following this, no additional studies were imputed through the trim and fill method. As a result, the research revealed that the summary mean effect was likewise robust to the risk of publication bias.

### Subgroup analysis and meta-regression

**Fig. 5:**
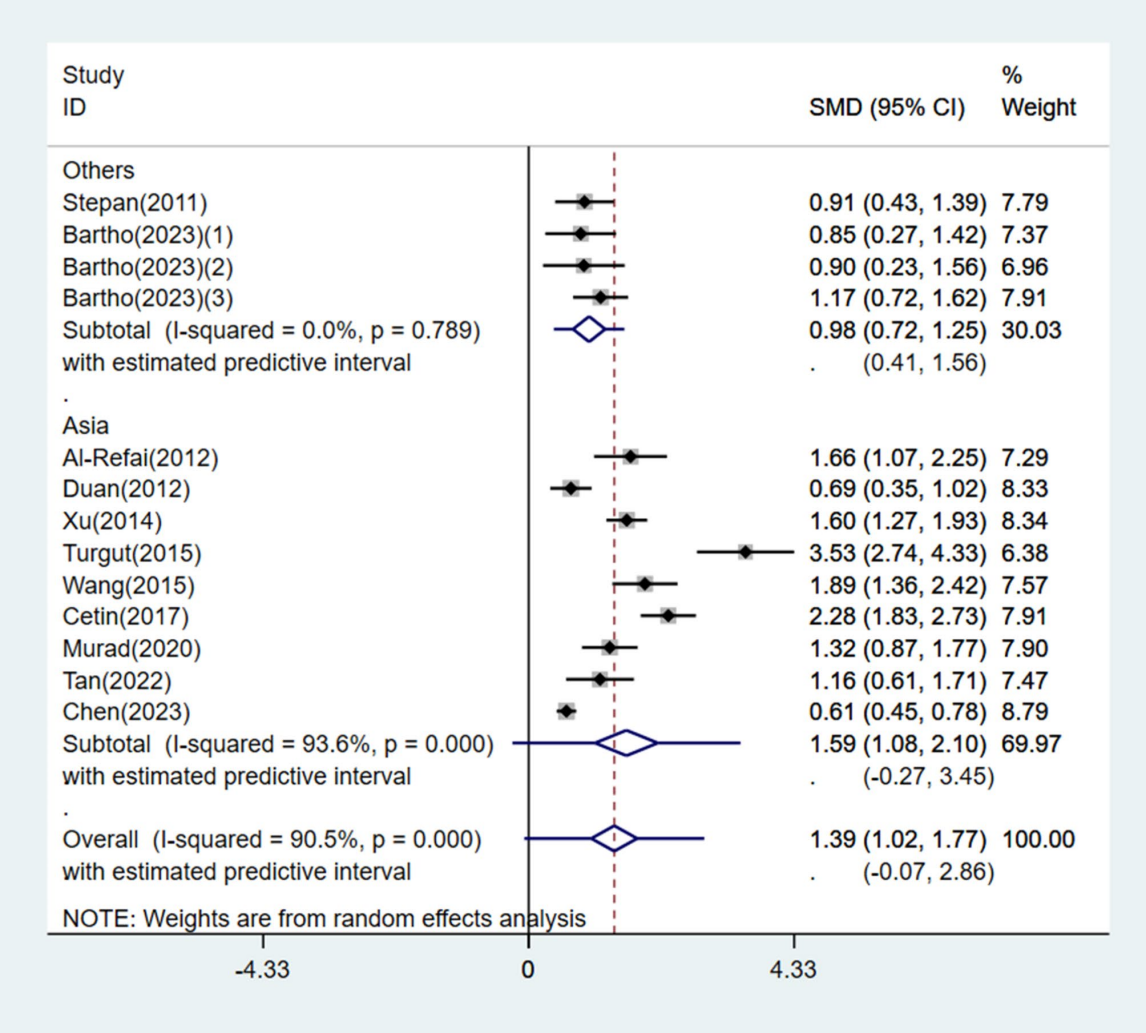
Subgroup analysis stratified by geographical location

**Fig. 6:**
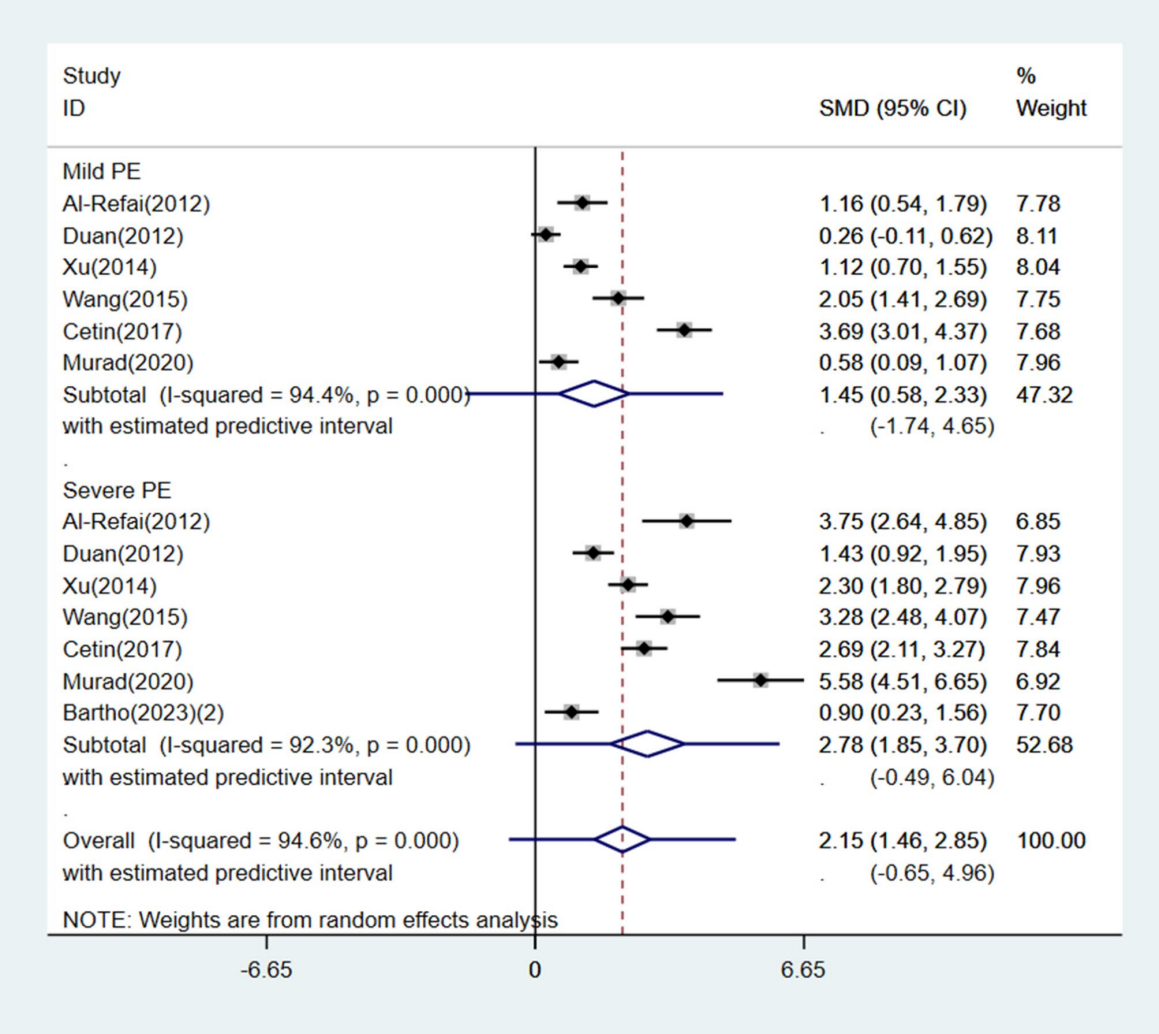
Subgroup analysis stratified by disease severity

**Fig. 7:**
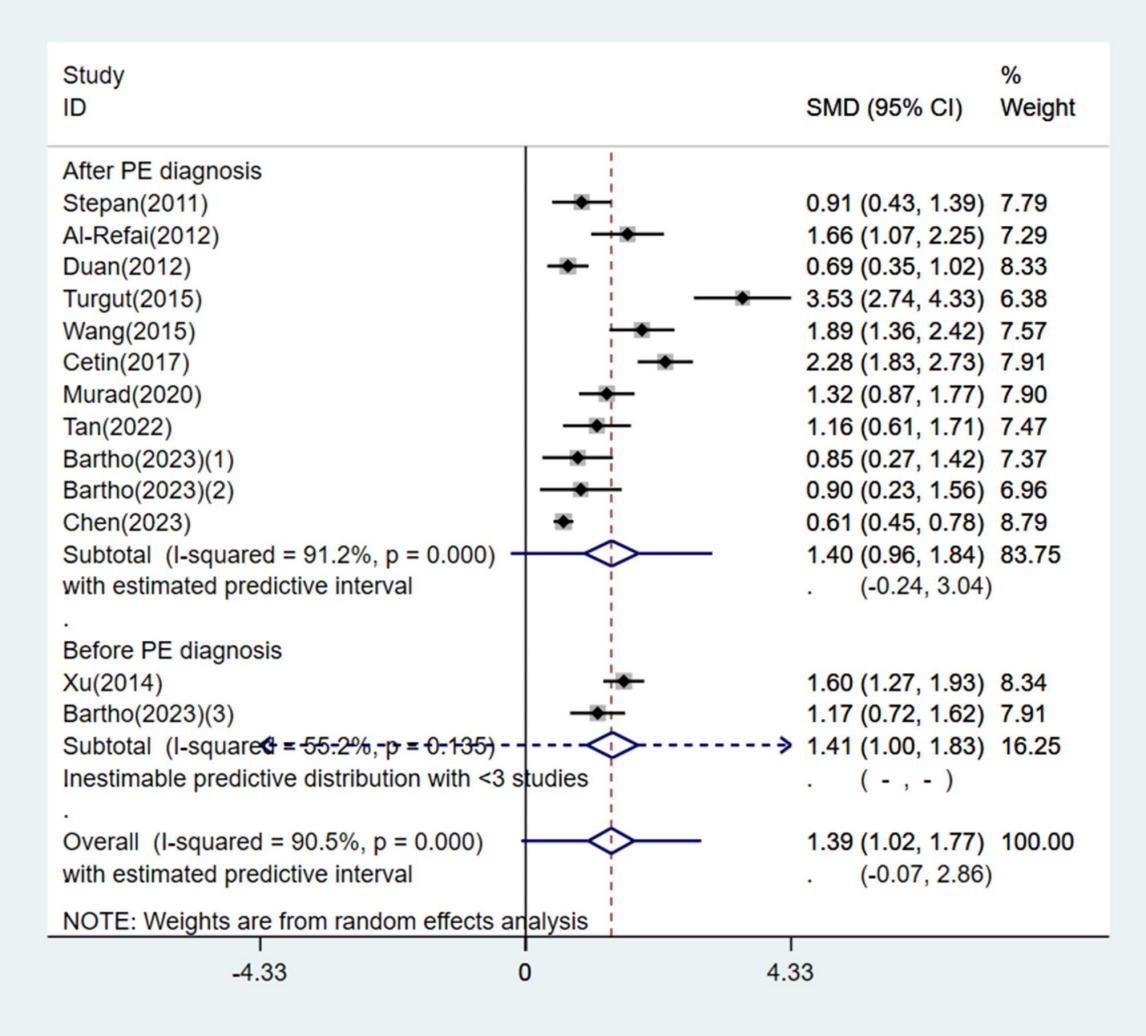
Subgroup analysis stratified by timing of sampling

**Fig. 8:**
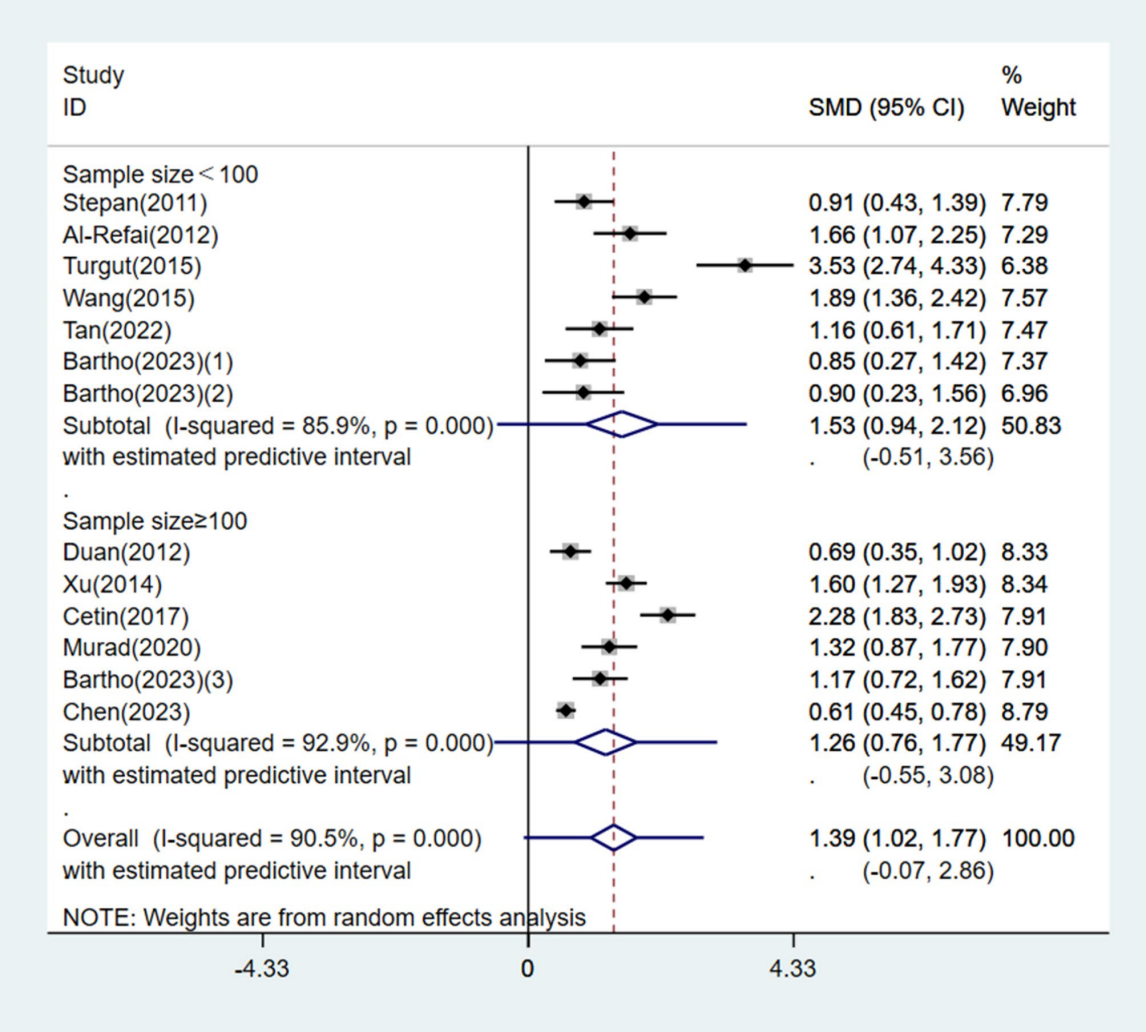
Subgroup analysis stratified by sample size

Subgroup analysis was performed based on geographical location, disease severity, timing of blood collection, and sample size. Circulating chemerin levels were significantly increased in both the Asian and non-Asian subgroups with PE compared with the normotensive group of pregnant women (Asia: SMD = 1.59, 95% CI: 1.08, 2.10, 95% PI: -0.27, 3.45, *I*^*2*^ = 93.6%, *P* < 0.001; Others: SMD = 0.98, 95% CI: 0.72, 1.25, 95% PI: 0.41, 1.56, *I*^*2*^ = 0.0%, *P* = 0.789) (Fig. 5). A total of 6 studies classified patients in the PE group as having either mild or severe PE, and one study included only severe PE patients. Subgroup analysis results stratified by disease severity also showed a significantly higher circulating chemerin level in the PE group (mild PE: SMD = 1.45, 95% CI: 0.58, 2.33, 95% PI, -1.74, 4.65, *I*^*2*^ = 94.4%, *P* < 0.001; severe PE: SMD = 2.78, 95% CI: 1.85, 3.70, 95% PI: -0.49, 6.04, *I*^*2*^ = 92.3%, *P* < 0.001) (Fig. 6). Moreover, subgroup analysis validated that circulating chemerin levels in the PE group were higher irrespective of the timing of sampling (i.e., before or after PE diagnosis) (before PE diagnosis: SMD = 1.41, 95% CI: 1.00, 1.83, *I*^*2*^ = 55.2%, *P* = 0.135; after PE diagnosis: SMD = 1.40, 95% CI: 0.96, 1.84, 95% PI: -0.24, 3.04, *I*^*2*^ = 91.2%, *P* < 0.001) (Fig. 7). For the subgroups with sample size < 100 and ≥ 100 participants, the circulating chemerin levels were signifcantly higher than those in healthy individuals (sample size < 100: SMD = 1.53, 95% CI: 0.94, 2.12, 95% PI: -0.51, 3.56, *I*^*2*^ = 85.9%, *P* < 0.001; sample size ≥ 100: SMD = 1.26, 95% CI: 0.76, 1.77, 95% PI: -0.55, 3.08, *I*^*2*^ = 92.9%, *P* < 0.001) (Fig. 8).

Regarding the subgroup analysis between PE patients and controls, the definite source of heterogeneity was regrettably not identified. Thereafter, a meta-regression was performed according to the location, timing of sampling, and total sample size; both failed to detect the source of heterogeneity (Table 2).

**Table 2:** Meta-regression analysis of the effects on preeclampsia patients and normal pregnancies

Covariates	Coefficients	Standard Error	95% CI	*P* value
**Sample size**	-0.734	0.505	(-1.876, 0.408)	0.180
**Timing of sampling**	0.653	0.665	(-0.851, 2.158)	0.351
**Location**	-0.956	0.500	(-2.087, 0.175)	0.088

CI: Confidence interval

## Discussion

The ongoing search for biomarkers for the early diagnosis and prediction of PE has garnered extensive attention [37]. Studies comparing the levels of circulating chemerin in PE patients and controls have grown in frequency during the past few years. Although previous studies have evaluated the role of adipokines in PE via systematic reviews or summarized the pathophysiology of chemerin levels in PE in narrative reviews, the present study is the first to investigate the relationship between circulating chemerin levels and PE via a meta-analysis [38, 39]. Despite all studies reporting higher circulating chemerin levels in the PE group, a meta-analysis is also needed, given that the confidence intervals of random-effects models tend to be wide, and positive findings may also be misinterpreted as negative findings [40]. Although the moderate to high methodological quality of the studies and the trim and fill method showed robust results, heterogeneity between studies remained high. Conversely, sensitivity analysis demonstrated that none of the studies significantly altered heterogeneity. Despite multiple subgroup analyses, the levels of heterogeneity remained high for most groups and the 95% PIs were wide for all measures, indicating high variability in the circulating chemerin levels in future studies.

It is reasonable to assume that the elevated circulating chemerin levels that were observed are attributable to a rise in chemerin synthesis. The chemerin level decreases after birth, indicating its placental origin [41, 42]. Circulating chemerin levels have been established to increase during pregnancy, exhibiting similar characteristics to other factors and adipokines known to favour proinflammatory conditions [43]. Unfortunately, the majority of studies were conducted over a wide sampling period, and hence, gestational weeks can only be extrapolated as a potential source of heterogeneity. It has been shown that chemerin is not only involved in the development of PE by affecting the migratory and invasive abilities of trophoblast cells but also promotes trophoblast pyroptosis and inflammation [44]. In addition, chemerin has been shown to be potentially implicated in endothelial cell-induced nitric oxide signalling [24]. Therefore, both adipose tissue and placenta are hypothesized to be essential sources of circulating chemerin and thus are implicated in the development of PE.

As is well documented, gestational weight gain during pregnancy and prepregnancy BMI are risk factors for PE [45]. High BMI predisposes the body to pathologies such as glucolipid metabolism disorders, inflammatory responses, and insulin resistance, which have also been reported to facilitate the development of PE [46]. Indeed, some studies did not include BMI-matched normal pregnancies; thus, this may also be a source of heterogeneity. However, Xu et al. [32] described that BMI and serum chemerin levels are independent predictors of PE. The stepwise regression analysis of another study showed that chemerin levels were not correlated with BMI [31]. Therefore, the relationship among BMI, serum chemerin levels, and PE remains to be elucidated.

Considering that only 3 studies reported the diagnostic value of circulating chemerin levels, no analyses of test accuracy were performed. In the only predictive study conducted in the first trimester, a serum chemerin level greater than 183.5 ng/mL was predictive of the risk of PE, with a sensitivity and specificity of 87.8% and 75.7%, respectively [32]. In addition, two other studies reported the diagnostic value of circulating chemerin after the diagnosis of PE. PE was diagnosed with 95.5% sensitivity and 95.7% specificity when the maternal serum chemerin level was greater than 252.0 ng/mL [25]. Another study reported that elevated serum chemerin levels (> 228.5 ng/mL) had a sensitivity of 89.3% and specificity of 94.1% for the diagnosis of PE; >380.9 ng/ml had a sensitivity of 93.9% and specificity of 100% for the diagnosis of severe PE [26]. As more multicentre studies with large samples become available in the future, the sensitivity and specificity of circulating chemerin levels can be further pooled to demonstrate its diagnostic and predictive value for PE.

### Strengths and limitations

The present study is the first to investigate the relationship between circulating chemerin levels and PE via a meta-analysis. In addition, this study covered a diverse population, and subgroup analysis showed similar expression patterns regardless of geographic location, severity, and timing of sampling The present study postulates that circulating chemerin levels are a promising potential biomarker for the prediction and diagnosis of PE. The current study also has some limitations that should be acknowledged. First, most of the included studies were retrospective, and no causal relationship could be inferred between circulating chemerin levels and PE. This study can only draw a conclusion about the correlation between chemerin and PE. If scholars need to further study whether chemerin is involved in the etiology and pathogenesis of PE, basic research and cohort study should be conducted. Second, the heterogeneity between studies was high, and neither subgroup analysis nor meta-regression was able to identify its sources. In the subgroup analyses regarding mild and severe PE, there was a mild reduction but still high heterogeneity between the two groups (*I*^2^ = 94.4% and 92.3%) compared to the overall heterogeneity (*I*^2^ = 94.6%), and caution should be taken in this result. The high heterogeneity could be due to the inherent differences in each participant that would affect their circulating chemerin levels according to the extent of adjustment for age, BMI, parity, and other confounding factors. It is possible that further inclusion of additional studies in the future may help to identify to sources of heterogeneity. Third, the gestational trimester of patients during blood sample collection was not reported in three studies, and hence the source of heterogeneity was not explored. Indeed, some studies reported prepregnancy BMI of pregnant women, whereas others reported the postpregnancy BMI; thus, the source of heterogeneity could not be determined. Last, the mean and SDs were estimated from the median, IQR, and range using the methods recommended by two research teams, which, despite being previously employed, could potentially have compromised these results [22, 23].

## Conclusions

The current meta-analysis highlighted that circulating chemerin levels are higher in pregnancies with PE than in normal pregnancies. Circulating chemerin levels were elevated in patients with PE even when measured before or after PE diagnosis. The above data supports the use of circulating chemerin as one of several potential biomarkers for future models that divide pregnancies into PE patients and health controls and predict the future progression of PE patients. However, the cause-and-effect relationship between chemerin levels and PE remains to be further investigated, with large-scale studies needed to determine the underlying potential molecular mechanisms and benefits of circulating chemerin levels in the prediction and diagnosis of PE, as well as to determine cut-off values for risk stratification in clinical practice.

### Electronic supplementary material

Below is the link to the electronic supplementary material.

Supplementary Material 1

Supplementary Material 2

Supplementary Material 3

## Data Availability

All data generated or analysed during the current study are included in this manuscript. Any further requests for data are available with the corresponding author.

## List of abbreviations

PE: preeclampsia
SMD: standardized mean difference
CI: confidence interval
PI: prediction interval
sFlt-1: soluble fms-like tyrosine kinase-1
PlGF: placental growth factor
BMI: body mass index
AUC: area under the curve
SD: standard deviation
IQR: interquartile range
